# The impact of a multi-component hospital avoidance programme in residential aged care homes: a stepped-wedge cluster randomised trial

**DOI:** 10.1093/ageing/afaf275

**Published:** 2025-10-06

**Authors:** Nicole M White, Xing J Lee, Michelle J Allen, Nicholas Graves, Gillian Harvey, Carla Shield, Trudy Dwyer, Claudia Meyer, Florin I Oprescu, Elizabeth V Cyarto, Jeffrey Rowland, Hannah E Carter

**Affiliations:** Australian Centre for Health Services Innovation and Centre for Healthcare Transformation, School of Public Health and Social Work, Queensland University of Technology, Kelvin Grove, Queensland, Australia; Australian Centre for Health Services Innovation and Centre for Healthcare Transformation, School of Public Health and Social Work, Queensland University of Technology, Kelvin Grove, Queensland, Australia; Australian Centre for Health Services Innovation and Centre for Healthcare Transformation, School of Public Health and Social Work, Queensland University of Technology, Kelvin Grove, Queensland, Australia; Duke-NUS Postgraduate Medical School, National University of Singapore, Singapore, Singapore; Australian Centre for Health Services Innovation and Centre for Healthcare Transformation, School of Public Health and Social Work, Queensland University of Technology, Kelvin Grove, Queensland, Australia; Flinders University College of Nursing and Health Sciences, Bedford Park, South Australia, Australia; Australian Centre for Health Services Innovation and Centre for Healthcare Transformation, School of Public Health and Social Work, Queensland University of Technology, Kelvin Grove, Queensland, Australia; Faculty of Health, Medicine and Behavioural Sciences, University of Queensland, Herston, Queensland, Australia; Appleton Institute, Central Queensland University, Rockhampton, Queensland, Australia; Bolton Clarke Research Institute, Forest Hill, Victoria, Australia; School of Primary and Allied Health Care, Monash University, Frankston, Victoria, Australia; School of Health, University of the Sunshine Coast, Sippy Downs, Queensland, Australia; Out Doors Inc, Kensington, New South Wales, Australia; Metro North Health, The Prince Charles Hospital, Chermside, Queensland, Australia; School of Nursing, Faculty of Health, Queensland University of Technology, Kelvin Grove, Queensland, Australia; Australian Centre for Health Services Innovation and Centre for Healthcare Transformation, School of Public Health and Social Work, Queensland University of Technology, Kelvin Grove, Queensland, Australia

**Keywords:** hospital avoidance, nursing home, health service use, emergency transfer, aged care, older people

## Abstract

**Objectives:**

To investigate if a multi-component hospital avoidance intervention would reduce hospital bed days in residential aged care (RAC) homes.

**Design:**

Prospective stepped-wedge cluster randomised trial with usual care and intervention phases.

**Setting:**

Eleven RAC homes in Queensland, Australia.

**Participants:**

The intervention targeted all nursing staff and personal care workers within the participating RAC homes. Outcome data were collected for all residents living in the participating RAC homes at any time throughout the trial period.

**Intervention:**

The intervention comprised four core components: face-to-face training sessions with all nursing staff and personal care workers; provision of diagnostic medical equipment; decision support tools and embedded implementation facilitation and support.

**Main outcome measures:**

The primary outcome was the number of hospital bed days per 100 resident days in RAC homes. Secondary outcomes assessed emergency department (ED) transfers, subsequent admissions to hospital and hospital length of stay.

**Results:**

No statistically significant intervention effects were observed across the reported outcomes. Exposure to the early detection of deterioration in elderly residents intervention was associated with a 27% relative increase in the primary outcome of hospital bed days (Estimate, 95% CI: 1.13, 0.93–1.74, *P*-value = 0.137). There was an 8% reduction in ED transfers (Estimate, 0.92: 0.74–1.14, *P*-value = 0.462) and a 10% increase in hospital admissions (Estimate, 1.10, 95% CI: 0.84–1.44, *P*-value = 0.486). For residents admitted to the hospital, the expected length of stay increased from 4.2 to 4.4 days (Estimate: 1.04; 95% CI: 1.00–1.07; *P*-value = 0.055).

**Conclusions:**

Whilst not statistically significant, findings indicate that the intervention was associated with fewer ED transfers, but increased hospital admissions and overall hospital bed days. Programme implementation was impacted by major contextual barriers, notably the COVID-19 pandemic, which contributed to pressures on staffing and workload.

**Trial registration:**

Australia New Zealand Clinical Trial Registry, ACTRN12620000507987 (registered 23^rd^ April 2020).

## Key Points

- Early detection of deterioration in elderly residents is a hospital avoidance programme for residential aged care home staff and residents.
- The programme was trialled in 11 aged care homes using a stepped-wedge cluster randomised trial design.
- No statistically significant intervention effects were observed on emergency transfer outcomes or length of stay.
- Implementation was impacted by major contextual barriers which contributed to pressures on staffing and workload.

## Introduction

People living in nursing or aged care homes are often frail and have been diagnosed with multiple comorbidities, placing them at risk of acute clinical deterioration [1]. These individuals may experience transfers to emergency departments (ED) [2, 3] which expose them to increased risk of adverse outcomes, including functional and cognitive decline, medical complications and fragmented care [4–6]. Access to acute care services is essential following an acute deterioration event to ensure that timely and appropriate care is provided in line with patient preferences. Whilst ED transfers and hospitalisation are often necessary for this population to address their complex medical needs, up to 60% of ED transfers [4] and up to two-thirds of hospitalisations [7] from aged care homes are potentially avoidable with appropriate early intervention. Evidence-based interventions to reduce potentially avoidable health service use in this population are therefore valuable to residents by reducing the risk of further deteriorations and to healthcare systems by easing demand for acute care services.

Despite these aforementioned risks, the current evidence for effective interventions is unclear. No single intervention component, or combination of components, has been found to consistently result in improvements in staff practice within residential aged care homes [8]. Evidence in favour of interventions to reduce unplanned health service use from aged care homes has primarily been generated from single-site or non-randomised study designs [9–15]. Evidence from multi-site randomised trials is comparatively limited [16, 17], and consisting of multi-component interventions tested using parallel cluster randomised designs. Published randomised trials have not reported statistically significant differences in health service use associated with tested interventions with possible explanations being logistical issues that may have diluted their clinical effectiveness. These issues reflect the limitations of parallel cluster randomisation when implemented in complex healthcare environments, including unexpected policy changes, suboptimal implementation strategies and short exposure times. Stepped wedge cluster randomised trials offer an alternative, pragmatic study design capable of balancing these and other logistical challenges with the robust evaluation of an intervention in routine healthcare settings [18].

The Early Detection of Deterioration In Elderly residents (EDDIE+) study was a stepped-wedge cluster randomised trial that implemented and evaluated the impact of a hospital avoidance programme for aged care home staff and residents. The EDDIE+ programme was a multi-component intervention that included staff education and training, clinical decision support, access to diagnostic medical equipment and local site facilitation [19]. It was previously evaluated as a single-site pilot study (EDDIE) that observed a 19% decrease in annual hospital admissions and a 31% decrease in average length of stay compared with usual care [9]. The development of EDDIE+ incorporated successful elements from the pilot study combined with implementation science strategies to implement the intervention across multiple sites.

The study we report on here trialled the EDDIE+ intervention in eleven residential aged care (RAC) homes in Queensland, Australia. In Australia, a RAC home provides long-term accommodation, personal care and health care for older persons who can no longer live independently at home, and is the preferred terminology in the Australian setting. The stepped wedge design enabled the intervention to be implemented in one RAC home at a time, allowing for consistent implementation of core intervention components and tailoring based on individual RAC home needs. Implementation, health service use and cost outcomes were evaluated before and after intervention exposure from 8 March 2021 to 1 May 2022. The study timeframe overlapped with Australia’s transition from pre-vaccination to post-vaccination settings, as part of the national COVID-19 response. Aged care staff and residents were the first population eligible for COVID-19 vaccination as part of this national response, from February 2021. The aim of this study was to evaluate the impact of EDDIE+ on health service use outcomes, compared with usual care in all participating RAC homes. The study hypothesis was that the EDDIE+ programme would reduce residents’ potentially avoidable hospital use by improving RAC home staff’s early recognition and management of resident deterioration.

## Methods

### Study design

**Figure 1:**
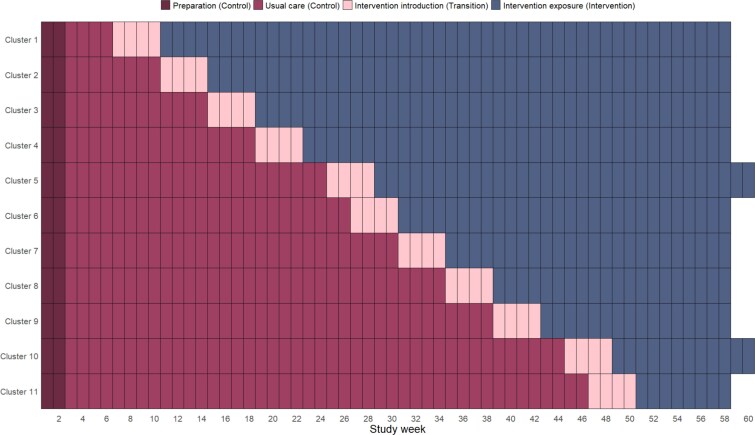
Stepped wedge cluster randomised trial design showing observed treatment sequences for implementing the EDDIE+ program. See *‘Study design’* for further details on observed treatment sequences for Clusters 5 and 10 and Control phase activities.

EDDIE+ was evaluated in 11 RAC homes over 60 weeks using a complete stepped wedge cluster randomised design (Figure 1, File S1). A single, not-for-profit aged care provider managed all RAC homes that participated in the study. The provider is Australia’s largest independent aged care provider with 99 RAC homes supporting 8718 resident places. RAC homes were purposively sampled to obtain a mix of metropolitan and non-metropolitan (‘regional’) sites, with a higher priority placed on homes without access to existing locally implemented hospital avoidance programmes and with higher bed capacity to ensure the trial was adequately powered [19].

Each RAC home was defined as a cluster, and each cluster was randomly allocated to a unique treatment sequence. Treatment sequences were defined by four-weekly time steps covering three distinct study phases: a Control phase during which usual care processes took place, a 4-week Transition phase to allow for initial implementation activities to be completed, and an Intervention phase. The duration of the Control and Intervention phases varied by assigned treatment sequence, up to 46 and 48 weeks, respectively (Table S1). All clusters started in the Control phase for the first 6 weeks of the study; the first 2 weeks comprised preparation activities across all clusters. Clusters were scheduled to start the Transition phase every 4 weeks thereafter. Initial activities completed during the Transition phase included site-specific context assessments and the development of local implementation plans [20].

Twelve RAC homes were initially enrolled in the study; four in regionally located areas and eight in metropolitan areas. One regionally located home withdrew after randomisation due to limited staff capacity. The 11 participating homes ranged in size from 91 to 164 beds, with staff numbers at trial commencement ranging from 13 to 25 nursing staff and 42 to 74 personal care workers. The Transition phase was delayed by 2 weeks for one cluster, and the Intervention phase was delayed by 2 weeks for another cluster after the study start date. This deviation from planned treatment sequences was within the acceptable parameters in the study protocol for managing local RAC home issues that could impact trial participation. The Intervention phase was extended by 2 weeks for both impacted clusters to compensate for these unplanned delays.

The trial was registered prospectively on the Australian and New Zealand Clinical Trials Registry on 23 April 2020 (ACTRN12620000507987). The protocol is available as an open-access publication [9].

### Participants

All residents living in participating Queensland RAC homes at any time during the study were eligible for inclusion. Our analysis used data on all residents transferred to a local hospital ED during the trial period. This analysis required resident-level data collected by the aged care provider to be linked with patient-level hospital records from state-level administrative data sources. Data linkage information is provided in the Supplementary Material (File S2).

### Ethics approval

Ethics approval to collect deidentified participant data was granted by the Residential Aged Care provider’s Research Ethics Committee (approval number: 170031), with administrative ethical approval granted by the Queensland University of Technology University Human Research Ethics Committee (2000000618). Ethics approval included a waiver of consent to access required RAC home and health services data for all outcomes presented in this paper. Approval to access hospital administrative data used in data linkage was granted under the Queensland Government Public Health Act (2005) (approval number 170031).

### Intervention

The control condition was defined as usual care policies and procedures for managing clinical deterioration amongst residents within individual RAC homes. The intervention condition was defined as the implementation of the EDDIE+ programme. The EDDIE+ programme aimed to provide RAC home nurses and personal care workers with the knowledge, confidence and resources required to identify early signs of deterioration in residents so they could initiate appropriate management in the residents’ home environment and reduce the likelihood of ED transfer and hospital admission.

Intervention components were defined by four core components, summarised in Table 1. Supplementary Table S2 provides additional information on the fixed and flexible elements of each component, and how these could be tailored to meet the needs of individual RAC homes. Context assessments were completed at each RAC home during the study’s Transition phase to inform site-specific implementation plans that preserved fixed elements of the intervention whilst allowing flexible elements to be adapted to support appropriate tailoring at each RAC home [20]. The intervention was delivered at the cluster level and maintained from the start of the intervention exposure until the end of the trial.

**Table 1:** EDDIE+ programme components.

EDDIE+ intervention component	Description
Education	Face-to-face training with all nursing staff and personal care workers, delivered by a nurse educator employed by the study. Training topics include identifying the early signs of clinical deterioration, proactive management and effective communication of deterioration concerns, including review and referral to attending General Practitioners to determine the need for ED transfer. Training sessions were delivered in the initial intervention establishment phase, with follow-up sessions provided throughout the intervention period on request as needed to capture newly commenced staff members.
	Education material toolkit covering mandatory content and additional materials based on individual home needs.^a^
Equipment	Diagnostic medical equipment provided to RAC homes based on an initial needs assessment, including bladder scanners, electrocardiogram machines, vital signs monitors and pulse oximeters.
	Simulation training and competency assessment delivered by the nurse educator to support appropriate equipment use by RAC home staff.
Decision support	Clinical decision-making aides developed to support the management of specific conditions linked with acute deterioration.
Implementation facilitation and support	Recruitment of an internal EDDIE+ facilitator from the local clinical team per RAC home, to support implementation through ongoing upskilling of staff, engagement with key stakeholders and data monitoring activities.
	Regular communication and ongoing support from the study-wide facilitator, the nurse educator and the RAC home leadership team.

^a^Education resources are available at https://www.aushsi.org.au/research/projects/eddie-resources/

### Outcomes

The primary outcome was the number of hospital bed days per 100 resident days. Resident days were estimated from reported resident numbers and available beds reported by individual RAC homes. Secondary outcomes assessed ED transfers, subsequent admissions to hospital and hospital length of stay. The number of hospital bed days, ED transfers and hospital admissions were defined per cluster-week. Hospital length of stay was defined per resident as the total number of days spent in hospital from the end of ED presentation until hospital discharge or in-hospital death.

### Sample size

The minimum sample size was determined by simulation [21] and was powered to detect at least a 41% reduction in the primary outcome [9]. This was based on a combination of a 19% reduction in hospital admissions and a reduction in average length of stay from 7.3 to 5.3 days, as observed in the pilot. The expected primary outcome under the Control condition was 346 hospital bed days per 100 residents per year based on the single-site pilot study. Data collection on 100 residents per RAC home based on planned treatment sequences returned an expected 91% power based on a two-sided alternative hypothesis with statistical significance defined at 0.05. Calculations assumed an intra-cluster correlation coefficient of 0.3.

### Randomisation

Study randomisation was performed using a random number generator with a set seed in the R statistical software by a trial statistician (XJL). Allocated treatment sequences were concealed from clusters until 10 weeks before the start of their Transition phase. Residents were blinded to the sequence allocation and were recruited continuously over the full study duration.

### Statistical methods

Outcome analyses treated data as cross-sectional to account for a mixture of new and existing residents at each RAC over the study timeframe. Analyses excluded ED transfers that occurred during a cluster’s Transition phase. Descriptive summaries for resident and transfer characteristics were calculated for Control and Intervention conditions. Summary statistics were aggregated across all clusters to preserve data confidentiality. Resident characteristics included patient age, sex and indigenous status. Transfer characteristics were the reason for ED transfer, arrival to ED by ambulance and ED length of stay. Elective status and care type were also summarised for residents admitted to hospital after ED transfer.

Primary and secondary outcomes were analysed using generalised linear mixed modelling (GLMM), assuming an exchangeable correlation structure and a random intercept per cluster. Intervention exposure was defined as a fixed effect, with a value of 0 for the Control condition and 1 for the Intervention condition. Time effects independent of intervention exposure were modelled as a linear fixed effect, using time in months since study commencement. For the primary outcome, the intraclass correlation coefficient (ICC) was estimated using a linear mixed model. The ICC is reported in this paper as an estimate of correlation between residents from the same cluster, to inform future sample size calculations [22].

The primary outcome was modelled as a Gamma distribution and was defined as total hospital days per cluster-week. Total resident days by cluster-week divided by 100 was included as a model offset. Intervention effectiveness was therefore modelled as the expected within-cluster change in total hospital bed days per 100 occupied resident days. Total ED transfers and hospital admissions were analysed per 1000 resident days, using a Poisson GLMM with a log link function. A Gamma GLMM analysed hospital length of stay with patient age in years and sex [Female (reference level), Male] included as additional fixed effects.

Model fixed effects were reported as point estimates with 95% confidence intervals. Estimates for total hospital days and hospital length of stay represented relative change in outcome. Reported effects for ED transfers and hospital admissions represented relative risks. Marginal predictions for each outcome were calculated to interpret the expected absolute change between Control and Intervention conditions.

Resident-level data linkage resulted in some ED transfers being excluded from analysis, due to a resident having either no corresponding hospital record or being matched to multiple hospital records based on available data fields.

All analyses were conducted in R version 4.0.0 using the tidyverse package for data processing, the lme4 package for GLMM estimation and the ggeffects package for marginal predictions by study phase.

As a sensitivity analysis, total hospital bed days and ED transfers were re-assessed using data from the aged care provider only without data linkage. Total hospital bed days for sensitivity analysis was defined as the total number of days between ED transfer and arrival back to the RAC home.

A de-identified dataset is available from the corresponding author upon reasonable request.

## Results

**Figure 2:**
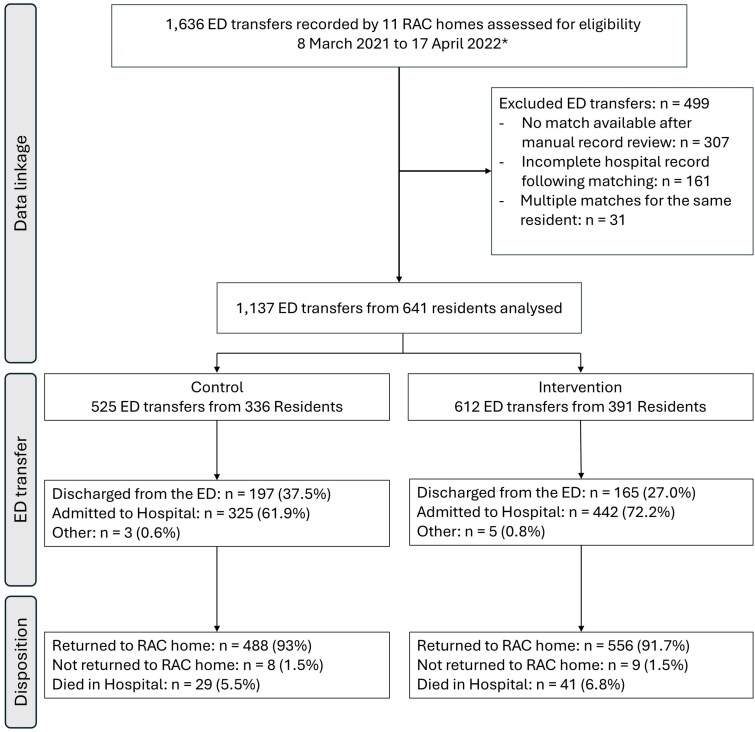
CONSORT study flowchart. *Two clusters were extended until 1 May 2022 to account for implementation delays. Further details are provided in *Study design*.

A total of 1636 ED transfers recorded were assessed for eligibility. Data linkage resulted in 1137 ED transfers from 641 residents, across all eleven clusters (69.5% of eligible participants) (Figure 2).

Eligible participants contributed 198 684 resident days in the Control phase and 245 710 in the Intervention phase (Table 2). The median resident age at emergency transfer was 86 years (Q1–Q3: 80–91 years). Approximately half (55%) of transferred residents were female. During the Control phase, 62% of ED transfers were admitted to hospital, compared with 72% of ED transfers in the Intervention phase. Hospital admissions were predominantly for acute care. Falls were the most documented reason for ED transfer and accounted for 46% of emergency transfers, followed by ‘other’. A post-hoc analysis of reasons for transfer within the ‘other’ category included acute deterioration events, loss of consciousness, general decline and wounds-related issues, all of which were more frequent in the intervention group. Other documented reasons included dyspnoea, which was more frequent in the control group (7.6%) compared to the intervention group (4.7%), and chest pain, which was slightly more common in the control group (5.1%) compared to the intervention group (3.6%). Transfers for constipation, delirium, dehydration and urinary tract infections were infrequent across study conditions. Observed outcomes across all 11 homes showed weekly variation with no clear trend before and after intervention exposure (Figure S1). Summary statistics and trial dates by RAC home are provided in the Supplementary Material (Table S3).

**Table 2:** Resident and ED transfer characteristics by study phase, unadjusted for clustering and time effects.

Characteristic	Control	Intervention
Total resident days	198 684	245 710
Resident characteristic		
Total residents with one or more ED transfers	336	391
Age, years	86 (80–91)	86 (80–91)
Female	178 (53%)	223 (57%)
Not Aboriginal or Torres Strait Islander	334 (99%)	380 (97%)
ED transfer characteristic		
Total ED transfers	525	606
Arrived by ambulance	502 (96%)	591 (98%)
ED length of stay, minutes	350 (220–574)	349 (227–524)
Admitted to hospital following ED transfer	325 (62%)	436 (72%)
Emergency admission	321 (99%)	430 (99%)
Acute care admission	322 (99%)	428 (98%)
Total hospital days	1494	1907
Hospital length of stay, days	2 (1–5)	2 (1–5)
Documented transfer reason		
Chest pain	27 (5.1%)	22 (3.6%)
Constipation	1 (0.19%)	6 (0.98%)
Dehydration	5 (0.95%)	4 (0.65%)
Delirium	5 (0.95%)	3 (0.49%)
Dyspnoea	40 (7.6%)	29 (4.7%)
Fall	243 (46%)	280 (46%)
Other	187 (36%)	253 (42%)
Palliative care	3 (0.57%)	0 (0%)
Urinary tract infection	14 (2.7)	9 (1.5%)

Exposure to the EDDIE+ intervention was associated with a 27% relative increase in the primary outcome that was not statistically significant (Estimate, 95% CI: 1.13, 0.93–1.74, *P*-value = 0.137; Table 3). This estimated change corresponded to an expected 5.98 (95% CI: 3.95–8.85) and 7.86 (4.11–14.34) hospital bed days per 100 resident days in the Control and Intervention phases, respectively. The estimated intra-cluster correlation coefficient was 0.09, which indicated a low correlation in the primary outcome amongst residents from the same RAC home (cluster).

**Table 3:** GLMM parameter estimates and predicted outcomes by study phase.

Outcome Model Parameter	Estimate (95% CI)	*P*-value	Predicted Outcome^a^ Estimate (95% CI)	
			Control	Intervention
Hospital bed days per 100 resident days (primary outcome)				
Intercept	0.94 (0.73–1.22)	–		
Intervention exposure	1.27 (0.93–1.74)	0.137	5.98 (3.95–8.85)	7.86 (4.11–14.34)
Study time (+1 month)	0.96 (0.93–1.00)	0.047		
Emergency transfers for 1000 resident days				
Intercept	2.69 (2.24–3.23)	–		
Intervention exposure	0.92 (0.74–1.14)	0.462	2.69 (2.24–3.23)	2.49 (1.85–3.34)
Study time (+1 month)	0.99 (0.97–1.02)	0.528		
Hospital admissions per 1000 resident days				
Intercept	1.77 (1.35–2.31)	–		
Intervention exposure	1.10 (0.84–1.44)	0.486	1.77 (1.35–2.31)	1.94 (1.32–2.86)
Study time (+1 month)	0.97 (0.94–1.00)	0.082		
Hospital length of stay				
Intercept	4.21 (4.06–4.36)	–		
Study time (+1 month)	1.01 (1.00–1.03)	0.068		
Intervention exposure	1.04 (1.00–1.07)	0.055	4.21 (2.92–6.06)	4.36 (2.51–7.58)
Patient age (+5 years)	0.96 (0.94–0.99)	0.008		
Sex: Male	0.88 (0.85–0.91)	<0.001		

^a^Prediction outcomes by study phase with other fixed effects set to zero/reference level. CI: Confidence interval.

Secondary outcome analyses indicated that the intervention was associated with a small reduction in ED transfers and a small increase in hospital admissions and length of stay after implementing the EDDIE+ programme (Table 3). Emergency transfers per 1000 resident days decreased by 8% from 2.69 in the Control phase, to 2.49 in the Intervention phase (Relative risk: 0.92; 95% CI: 0.74–1.14; *P*-value = 0.462). Following emergency transfer, hospital admissions increased by 10% from 1.77 to 1.94 admissions per 1000 resident days. For residents admitted to the hospital, the expected length of stay increased from 4.2 to 4.4 days (Estimate: 1.04; 95% CI: 1.00–1.07; *P*-value = 0.055) after adjusting for age and sex. Estimated intervention effectiveness across all outcomes was not statistically significant.

Sensitivity analysis of all transfers, irrespective of data linkage between RAC home and state administrative data sources is summarised in Table 4. Results indicated a 47% relative increase in the primary outcome in the Intervention phase (Estimate: 1.47; 95% CI: 1.09–1.98; *P*-value = 0.012). Compared with results based on linked hospital records only, predicted hospital bed days were higher before and after the EDDIE+ programme was implemented. These differences are likely attributable to differences in residents’ hospital discharge dates and RAC home return dates, as hospital discharge dates were unavailable for unlinked admissions; RAC home return dates were therefore used as a proxy. For example, residents who were discharged from either the ED or hospital may have been transferred to another healthcare facility for follow-up care prior to returning the RAC home. Analysis of emergency transfers was generally consistent with the main analysis. However, predicted outcomes were slightly higher in both study phases due to more transfers being included.

**Table 4:** GLMM parameter estimates and predicted outcomes by study phase from sensitivity analysis. CI: Confidence interval.

Outcome Model Parameter			Predicted Outcome Estimate (95% CI)	
	Estimate (95% CI)	*P*-value	Control	Intervention
Hospital bed days per 100 resident days (primary outcome)				
Intercept	1.20 (0.93–1.55)	–	7.95	12.13
Intervention exposure	1.47 (1.09–1.98)	0.012	(5.57–11.18)	(7.01–20.52)
Study time (+1 month)	0.95 (0.91–0.98)	0.002		
Emergency department transfers for 1000 resident days				
Intercept	3.32 (2.77–3.97)	–	3.32	3.22
Intervention exposure	0.97 (0.79–1.18)	0.756	(2.78–3.97)	(2.44–4.25)
Study time (+1 month)	0.98 (0.96–1.01)	0.150		

## Discussion

This study has presented findings on the effectiveness of a hospital avoidance programme (EDDIE+) implemented in 11 RAC homes as a stepped-wedge cluster randomised trial. Our analysis of primary and secondary outcomes found that the EDDIE+ programme was associated with small changes in ED transfers and hospital admissions compared with usual care. Intervention effectiveness estimated from our main analysis was not statistically significant, with results indicating that outcomes decreased over time independent of intervention exposure. Sensitivity analysis suggested a statistically significant increase in hospital bed days following intervention exposure.

Whilst the intervention group demonstrated a slightly lower proportion of ED transfers for certain conditions, such as dyspnoea and chest pain, there was an increase in transfers for ‘other’ documented reasons. A reduction in palliative care-related transfers in the intervention group was also observed. Possible explanations for these unexpected findings are that the intervention influenced the type of cases resulting in ED transfer, or reflected a shift in care delivery towards managing end-of-life care within the residential setting. However, these possible explanations are speculative, as the trial was designed and powered to examine changes in all ED transfers, irrespective of the reasons for transfer.

The observed lack of effectiveness of EDDIE+ in reducing hospital bed days contradicts findings from the evaluation of an earlier pilot programme which reported a decrease in both hospital admission rates and average length of stay per admission [9]. The pilot evaluation was conducted as a single-site, observational study and may have been subject to design-related bias. Interestingly, a similar conflict between pilot and multi-site findings was documented in published evaluations of the INTERACT (Interventions to Reduce Acute Care Transfers) programme that aimed to reduce avoidable ED transfers and hospital admissions amongst RAC residents in the United States.

The INTERACT programme was not related to EDDIE+ but adopted similar approaches, particularly in targeting early detection of deterioration through improved communication and decision support tools. Whilst a non-randomised pilot study of the INTERACT programme reported reduced rates of hospitalisations [15], a subsequent multi-site parallel cluster randomised trial did not find a statistically significant result [17].These findings highlight the challenges of implementing system-level change in complex settings, particularly when programmes are scaled across multiple diverse sites. This is supported by findings from the EDDIE+ process evaluation which reported that, despite widespread acceptance and support from staff, there was substantial variation in engagement and intervention fidelity across the 11 participating RAC homes [23]. Key barriers to fidelity included workforce shortages which contributed to high turnover and vacancies in internal facilitator roles. This led to inconsistencies in how the internal facilitator role was applied across homes. Workforce related barriers were further exacerbated by wider contextual factors including COVID-19 and local environmental events.

A key strength of this study was the adoption of a rigorous stepped wedge cluster randomised design. Whilst such trials are an increasingly popular pragmatic study design in health services research, their use in residential age care homes has remained limited, reflecting the broader challenges of conducting trials in these settings [24, 25]. Compared with other randomised designs, stepped wedge designs are characterised by the sequential roll-out of an intervention at the cluster level, where each cluster serves as its own control. This feature is particularly appealing for interventions targeting improvements in health service delivery, as all clusters can benefit from the intervention. A related strength of this design that benefitted the EDDIE+ study was the ability to introduce the intervention to one RAC home at a time, which helped to maximise consistency in the delivery of implementation activities whilst also allowing sufficient time to tailor the intervention to suit local contexts [20]. Time-sensitive recruitment, cluster retention and data collection burden are common challenges associated with stepped-wedge designs, that should be considered carefully at the planning stage [26]. These challenges were experienced by the EDDIE+ study, noting that one RAC home withdrew before the intervention was introduced and was replaced by a reserve site. Whilst this study used existing administrative data to reduce data collection burden, the trade-off with this decision was an inability to match all ED transfers reported by RAC homes to ED presentations and hospital admissions reported in state-level administrative databases. Recent advances in stepped wedge designs that accommodate delays in recruitment [27] and reduce the number of cluster-periods for data collection [28] provide alternative approaches to addressing these challenges in future studies.

Despite being a pragmatic design, the time-dependent nature of stepped wedge trials also poses risks when evaluating healthcare-based interventions. The EDDIE+ study commenced in March 2021 and was one of two stepped wedge trials run by our research group during the COVID-19 pandemic [29]. The burden of COVID-19 on Australia’s healthcare system was relatively low compared with other countries, in part due to national policies implemented in early 2020. Whilst these strategies were largely successful in reducing community transmission, reported COVID-19 incidence amongst RAC home residents during the first year of the pandemic was 10 times higher than the general Australian population [30]. Successive lockdowns during 2021 in response to outbreaks and to reduce residents’ exposure to community transmission may have resulted in fewer ED transfers for non-urgent care [31, 32]. Our analysis supports this possibility, as results estimated a general downward trend in ED transfers over the study timeframe after adjusting for intervention exposure. This finding correlates with observational studies that reported decreases in ED presentations and hospitalisations during the COVID-19 pandemic, which were most pronounced before vaccines became available [32–36]. More broadly, qualitative analysis of pragmatic clinical trials conducted overseas during the COVID-19 pandemic highlighted several implementation and statistical challenges, particularly for stepped wedge trials, due to delayed or paused intervention rollout [37]. Despite these challenges, our trial successfully achieved its planned sample size and minimised unexpected delays. We recommend that the limitations posed by the COVID-19 pandemic be considered when contextualising findings in other health systems and non-pandemic settings.

Primary outcome results indicated an increase in total hospital bed days per 100 resident days, which was statistically significant in sensitivity analysis based on aged care provider records only. When interpreted in conjunction with the interventions’ impact on ED transfers and average length of stay, a potential explanation for this result is that residents admitted to hospital during the Intervention phase were more complex and required longer-term care. However, it was not possible to determine the relative impact of patient case-mix factors within the available data, and this warrants further investigation.

Data collected during the trial showed that Falls were the most documented reason for ED transfer, consistent with longer hospital stays observed during the Intervention phase [38]. Falls have been estimated to account for 20% of healthcare expenditure in RAC homes, and ⁓60% of residents experience at least one fall incident [39]. Despite this burden, previous research on risk reduction interventions has highlighted significant challenges in reducing fall risk amongst this frail population [40]. In addition, national policy directives [41] require any resident whose fall resulted in a head injury to be transferred to hospital regardless of the extent of injury, further impacting on the ability to reduce transfer rates [38]. Whilst the EDDIE+ study was not powered to detect changes in health service outcomes based on different reasons for ED transfer, our findings may help to refine target areas for future hospital avoidance programmes.

RAC homes operate under three distinct provider types in Australia: publicly funded, privately funded and not-for-profit. The not-for-profit sector operates the majority of RAC homes in Australia, accounting for 56% of places. The EDDIE+ study was conducted in partnership with Australia’s largest not-for-profit aged care provider, and all homes were located within the state of Queensland. The use of purposive sampling approach ensured that included sites were representative of both metropolitan and non-metropolitan areas. Findings may be generalisable to other not-for-profit operated RAC homes in Queensland, and potentially more broadly within similar organisational contexts.

A mixed methods process evaluation, informed by the integrated-Promoting Action on Research Implementation in Health Services (i-PARIHS) framework, was conducted alongside the EDDIE+ trial and the detailed results are reported in a separate paper [23]. The process evaluation studied implementation outcomes including the fidelity and acceptability of the EDDIE+ programme and barriers and enablers of implementation. Data were collected from documentation completed as part of the study, staff self-efficacy surveys and interviews with RAC staff in participating homes. Additional interviews were conducted with family members of residents and the project team staff involved in delivering and supporting the intervention.

Findings highlighted a high level of acceptability of the EDDIE+ programme, with staff reporting increased knowledge, skills, confidence, competence and communication in relation to managing clinical deterioration. However, implementation was challenged by significant contextual barriers outside the control of the organisation and the project team, notably the COVID-19 pandemic, which placed significant pressures on staffing and workload. In turn, this influenced the ability of homes to maintain fidelity of implementation, for example, in relation to planned delivery of education sessions, enactment of the clinical facilitator role and use of the additional equipment provided as part of EDDIE+. These challenges are consistent with Australian and international evidence of the pervasive impacts of COVID-19 related interruptions to RAC care delivery [42, 43].

In conclusion, the EDDIE+ intervention did not result in statistically significant improvements in primary and secondary outcomes. Findings indicate that the intervention was associated with fewer ED transfers, but increased hospital admissions and overall hospital bed days. We recommend that future hospital avoidance studies in the RAC setting attempt to address or mitigate issues relating to high staff turnover and workload pressures, which were key barriers to implementation.

## Supplementary Material

aa-25-1057-File004_afaf275

aa-25-1057-File005_afaf275
